# Detection of Feline Coronavirus in Feline Effusions by Immunofluorescence Staining and Reverse Transcription Polymerase Chain Reaction

**DOI:** 10.3390/pathogens9090698

**Published:** 2020-08-25

**Authors:** Yi-Chen Luo, I-Li Liu, Yu-Tan Chen, Hui-Wen Chen

**Affiliations:** 1 Department of Veterinary Medicine, National Taiwan University, 10617 Taipei, Taiwan; iisabel1234tw@gmail.com (Y.-C.L.); yutanc2001@gmail.com (Y.-T.C.); 2 Institute of Veterinary Clinical Science, School of Veterinary Medicine, National Taiwan University, 10617 Taipei, Taiwan; liuili@ntu.edu.tw

**Keywords:** feline coronavirus, immunofluorescence staining, genotyping, spike protein gene, phylogenetic analysis

## Abstract

Feline coronavirus (FCoV), the pathogen for feline infectious peritonitis, is a lethal infectious agent that can cause effusions in the pleural and abdominal cavities in domestic cats. To study the epidemiology of FCoV in Taiwan, 81 FIP-suspected sick cats with effusive specimens were recruited to test for FCoV infection using immunofluorescence staining and reverse transcription-polymerase chain reaction as detection methods, and viral RNAs were recovered from the specimens to conduct genotyping and phylogenetic analysis based on the spike (S) protein gene. The results revealed that a total of 47 (47/81, 58%) of the sick cats were positive for FCoV in the effusion samples, of which 39 were successfully sequenced and comprised of 21 type I strains, 9 type II strains, and 9 co-infections. The signalment analysis of these sick cats revealed that only the sex of cats showed a significant association (odds ratio = 2.74, 95% confidence interval = 1.06–7.07, *p* = 0.03) with the infection of FCoV, while age and breed showed no association. FCoV-positive cats demonstrated a significantly lower albumin to globulin ratio than negative individuals (*p* = 0.0004). The partial S gene-based phylogenetic analysis revealed that the type I strains demonstrated genetic diversity forming several clades, while the type II strains were more conserved. This study demonstrates the latest epidemiological status of FCoV infection in the northern part of Taiwan among sick cats and presents comparisons of Taiwan and other countries.

## 1. Introduction

Feline coronavirus (FCoV) is an enveloped, positive-sense, single-stranded RNA virus of the genus *Alphacoronavirus*, subfamily *Orthocoronavirinae*, family *Coronaviridae* within the order *Nidovirales* [1,2,3]. FCoVs are comprised of two pathogenic biotypes: Feline enteric coronavirus, associated with mild enteric infections, and feline infectious peritonitis (FIP) virus, causing a lethal immune-mediated disease [4]. Though very different, no genomic differences between the two biotypes have been found [5]. Additionally, the subtle onset and varying clinical forms of FIP—the wet form, displaying exudative fibrinous serositis in the pleural or abdominal cavity, and the dry form, with disseminated perivascular pyogranuloma emerging in several organs [2,6,7]—have made clinical diagnosis difficult.

Traditionally, FIP diagnosis has mostly relied on histopathological examination and positive immunohistochemistry within lesions, but currently, detecting an FCoV antigen within an effusion macrophage can also be a good approach for viral antigen detection [8,9], and those involved mostly choose to target the nucleocapsid (N) protein due to its high-yield production in infected cells [10,11]. In addition, gene detection has also been reported to be an efficient method for viral detection. Reverse transcription-polymerase chain reaction (RT-PCR) tests targeting viral spike (S) genes [12] and untranslated region (UTR) [13] genes have previously been reported. While UTRs are more conserved regions that serve perfectly for viral detection, the S gene is considered to be more divergent, since the protein is responsible for the two serotypes [14]. Therefore, beside detection, the S gene could be used for genotyping tests, as well to conduct epidemiological research [5,15,16]. Since both tests are less aggressive and could be easily conducted ante-mortem, both RT-PCR and immunostaining of the FCoV antigen are commonly performed for the detection of viral existence [9].

Furthermore, many epidemiologic studies have been conducted in different countries to study the genotype prevalence and the factors that trigger disease development. FCoV can be divided into two genotypes—type I and type II [3]—based on structural differences in the spike protein, which is known to be related to the entry of the virus [2]. Regardless of the detection method, type I FCoV appears to be the dominant genotype in most countries, including Korea (3.8%) [5], Japan (83.3%) [17], China (95.8%) [16], the United Kingdom (97%) [12], Austria (86%) [18], and Switzerland (68%) [14]. However, on the other hand, studies on risk factors are not as consistent as those on the genotype prevalence. Whereas some studies have suggested sex as a factor that affects the development of FIP, others have suggested that age and breed are correlated factors [8,17]. The purpose of this study is to provide an outline of the FCoV status of Taiwan using an indirect immunofluorescence assay (IFA) and RT-PCR as detection methods, and to search for potential factors associated with the infection of FCoV or the potential for the development of FIP by analyzing the signalments of sick cats. The S gene sequences isolated from FCoV-infected cats were also analyzed and compared with reference sequences.

## 2. Results

### 2.1. Detection of FCoV and Signalment Analysis

**Figure 1:**
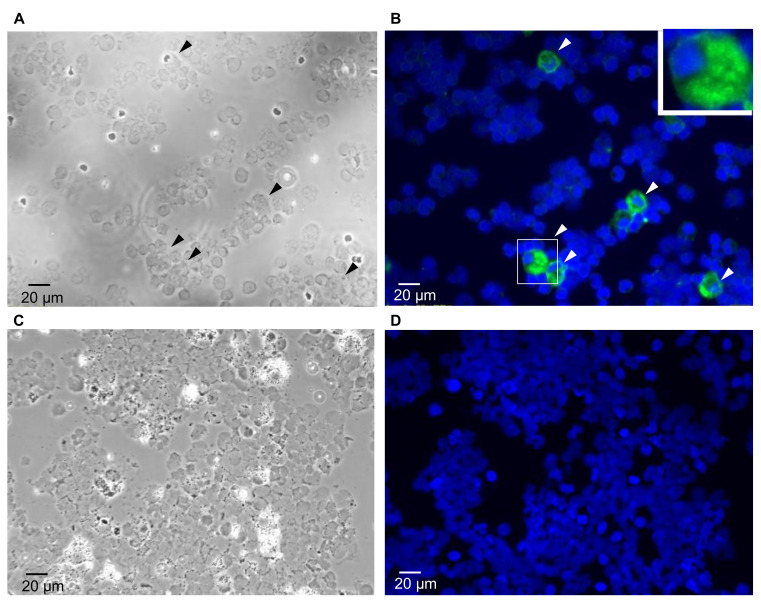
Indirect immunofluorescence staining for feline coronavirus (FCoV) in effusion samples. (**A**) (**C**) View of macrophages under the phase-contrast microscope. (**B**) Macrophages with positive fluorescence (FITC, green) signals (arrowed) with the reference of nucleus staining (DAPI, blue). (**D**) Macrophages presenting no cytoplasmic fluorescence signals with the reference of nucleus staining. Bar = 20 µm. Magnification: 400×.

A total of 81 samples (including pleural effusion, ascites, tumor fluid, and renal subcapsule effusion) was collected from FIP-suspected cats between September 2017 to January 2019, all of which were tested with IFA and RT-PCR. In a representative case, granular cytoplasmic fluorescence with a sharp hollow that represented the nucleus of the macrophage (Figure 1A,B, arrowed) was interpreted as a positive IFA view for FCoV detection. On the contrary, as indicated in Figure 1C,D, the negative FCoV detection presented no cytoplasmic fluorescence signals in the macrophages.

**Figure 2:**
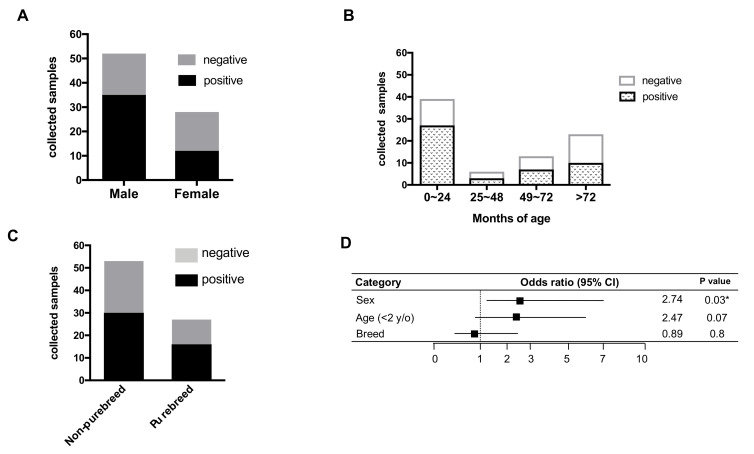
Association factor analysis. The results of FCoV detection within the body effusion for different (**A**) sexes, (**B**) months of age within four different age intervals, and (**C**) breeds are shown. (**D**) Odds ratios and the 95% confidence interval (CI) were calculated for each factor recorded from the cats to identify the association factor for FCoV detection. Fisher’s exact test was used for obtaining the *p* values.

In total, 53 out of the 81 cats (53/81, 65.4%) were positive for FCoV detection using IFA, while the other 28 cats were negative. However, to avoid obtaining false positive results by IFA, RT-PCR results were also used as a confirmation method. Among the 53 IFA positive samples, six were negative in both sets of RT-PCR tests, which targeted the 3′UTR and spike gene, respectively. Therefore, combining the IFA and RT-PCR results, we detected 47 (47/81, 58%) FCoV-positive samples and the sex, age, and breed of cats are shown in Table 1. The positive rates of FCoV in male and female cats’ body effusion macrophages were 67% (35/52) and 43% (12/28), respectively, with missing sex data for one FCoV-negative cat (Figure 2A). The positive rates of cats in different age intervals (0–24, 25–48, 49–72, and >72 months old, with age ranging from 2 months to 228 months) were 69% (27/39), 50% (3/6), 54% (7/13), and 43.4% (10/23), respectively (Figure 2B). The positive rates of purebred cats, including 10 different breeds, and non-purebred cats were 32% (17/53) and 66% (35/53), respectively, with missing data for one FCoV-positive cat (Figure 2C). The detection of FCoV within body effusion macrophages was significantly associated with the sex of the cats (odds ratio (OR) = 2.74, 95% confidence interval (CI) = 1.06 to 7.075, *p* = 0.03), but was not significantly correlated with age (OR = 2.47, 95% CI = 0.9–6.1, *p* = 0.07) or breed (OR = 0.89, 95% CI = 0.35–2.2, *p* = 0.8) (Figure 2D).

**Figure 3:**
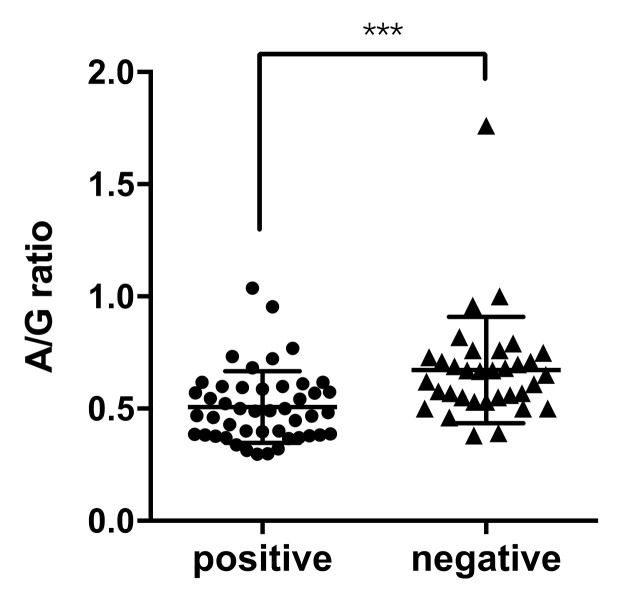
Comparison of the A/G ratio in cats with FCoV-positive or FCoV-negative. A/G ratios from sampled cats are shown. Data were expressed as the mean ± s.d. An unpaired Student’s *t*-test was performed to compare the two groups. *** *p* < 0.001.

The albumin to globulin (A/G) ratio blood test for these clinically suspected cats was also recorded. The 47 positive cats, with missing data for one cat, exhibited a significantly (*p* = 0.0004) lower value of the A/G ratio (mean = 0.51, ranging from 0.29 to 1.03) than other 34 negative cats (mean = 0.67, ranging from 0.38 to 1.76) (Figure 3).

### 2.2. Genotyping and Phylogenetic Analysis of the FCoV Strains

**Figure 4:**
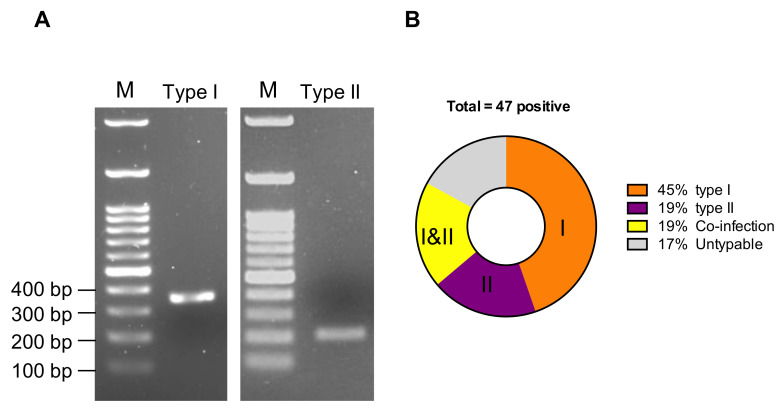

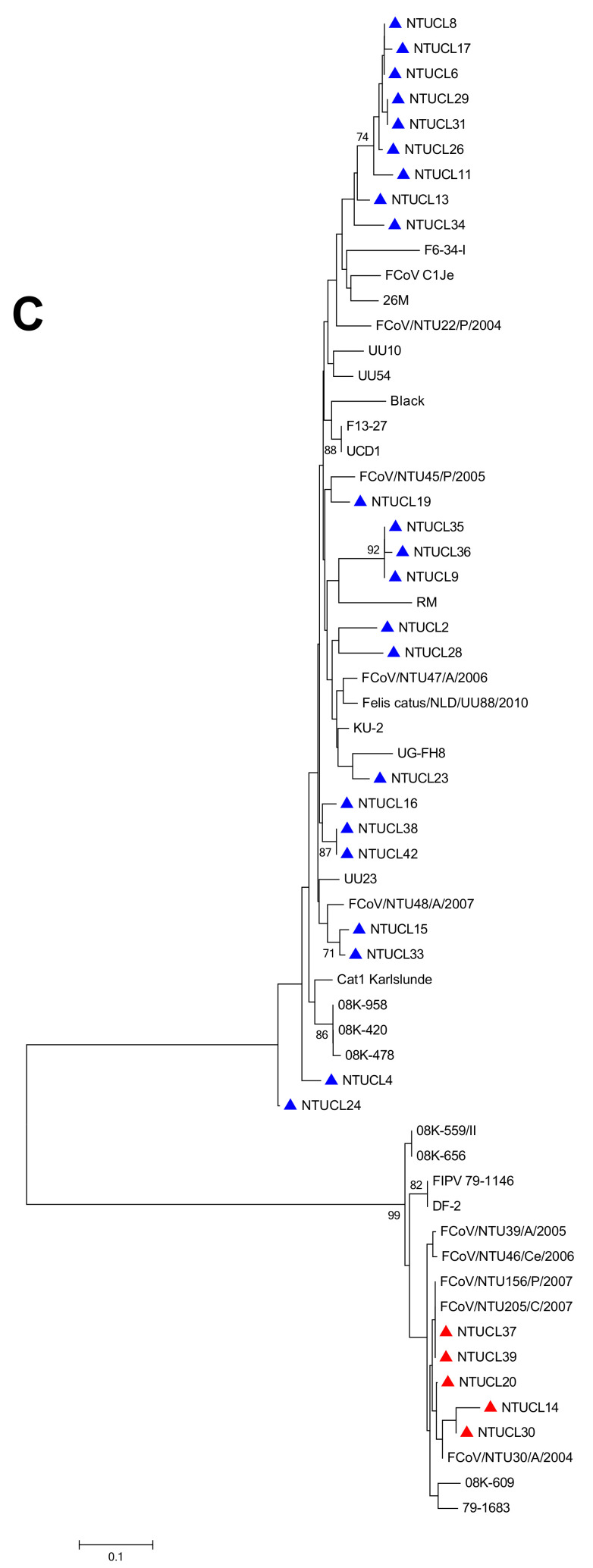
Viral genotyping and phylogenetic analysis based on the partial spike gene. (**A**) Representative gel images of genotyping RT-PCR. M: marker. (**B**) The results of the FCoV genotyping in this study are shown, including type I (orange patch), type II (purple patch), co-infection (yellow patch), and untypable (gray patch) groups. (**C**) All isolated type I (blue triangle) and type II (red triangle) strains were included in the phylogenetic analysis and compared with reference strains to construct a phylogenetic tree. The tree was plotted with MEGA 6.0 using the neighbor joining method (bootstrapping for 1000 replicates with its value >70%).

Figure 4A demonstrates representative results of the genotyping PCR. Type I strains yielded a 360-bp amplicon, and type II strains yielded a 218-bp amplicon in gel electrophoresis. Samples diagnosed as positive in the UTR-based PCR but that gave negative results in the genotyping PCR were recorded as untypable, and samples demonstrated positive for both type I and type II PCR were recorded as co-infection. Among the 47 positive samples, 21 (45%, 21/47) were grouped as type I strains, 9 (19%, 9/47) were grouped as type II strains, 9 (19%, 9/47) were co-infected with both types, and 8 (17%, 8/47) were untypable (Figure 4B). PCR products of all 39 typable samples were submitted for sequencing, from which 28 assured sequences were obtained and further analyzed. The nucleotide sequences of the partial S gene reported in this study have been submitted to the GenBank database and have been assigned accession numbers from MK736783 to MK736810. Multiple alignment revealed that Taiwan type I and type II FCoV isolates yielded nucleotide identity values from 83.2 to 100% and 97.4 to 100%, respectively, while the amino acid analysis yielded identity values from 82.9 to 100% and 97.4 to 100%, respectively. Furthermore, all typable sequences were selected to develop a phylogenetic tree that compared them with reference strains (Figure 4C). According to the phylogenetic tree, type I and type II strains could be clearly delineated, and while our type I isolated strains could be further subdivided into several small clades and grouped with strains from other countries, our type II isolated strains only aggregated into one clade.

## 3. Discussion

In Taiwan, FIP has been an emerging problem for the rising number of domestic cats in recent years. However, few epidemiological studies concerning FIP or FCoV have been conducted in Taiwan in recent years. This is because diagnosing FIP can be difficult due to its varying symptoms at different stages and in different forms Therefore, instead, we turned to an indirect immunofluorescence assay and RT-PCR for FCoV detection. Though convenient, it is worth keeping in mind that none of the detection methods are good enough to completely exclude a histopathological examination [9], but including both tests could compensate for each of their disadvantages and give a more confident result of detection. Therefore, in this study, a sample was only viewed as positive when it tested positive for both IFA and RT-PCR, targeting either 3′UTR or spike gene primers. In our study, 81 body effusion fluid samples from sick cats with body effusion were included for FCoV detection, and the positive rate of these samples was 58% (47/81), which was much higher than those of other Asian countries, including Korea (19.3%), Japan (44.1%), and Turkey (37%) [5,19,20]. Despite this, the rate is lower than that of China (74.6%) in 2018 [16] and that reported in Taiwan 10 years ago [21]. The discrepancy might arise from the differences in sensitivity between different detection methods or indicate a rather high prevalence of FCoV in east Asia, since higher detection rates have mostly been obtained in China and Taiwan. In addition, the differences in clinical criteria for diagnosis of sick cats with body effusions may contribute to differences in observed prevalence.

In several previous studies, the signalments, including the sex, age, and breed, of cats were recorded and analyzed; however, no universal agreements about the correlation between a signalment and disease development have yet been reached. Though our data could not be extrapolated to the prevalence of FIP for all of Taiwan, our result has given us an insight into the FCoV infection situation among sick cats. While studies in Australia [22] and Malaysia [23] have reported that there is no correlation between age and FCoV infection, which is in line with our results, other studies have reported an association between cats younger than two years old and FCoV infection [8,24]. Furthermore, our current study showed no significant correlation between breed and FCoV infection, which is in line with studies in Turkey [20] and China [16]. Comparatively, most studies have reported that some specific breeds of cat, such as Persian in Malaysia [23] and British short hair, Devon Rex, and Abyssinian in Australia [8], seem to have a stronger tendency toward the development of disease due to FCoV. In contrast, whereas other factors showed no relationship with FCoV infection, sex was the only association factor of our study that showed a significant correlation (*p* = 0.03), which is consistent with studies conducted in Australia [8,25] and America [24] demonstrating that male cats show a greater tendency toward infection of FCoV. In addition, previous reports have suggested that there might be several other predisposing factors, such as behavior, hormones, and stress, that might indirectly cause male cats to show a stronger tendency toward the development of FIP [26], which supported our study results.

The limitation of this study is that we used a convenient sampling method; therefore, our results cannot represent the overall situation of Taiwan, for we have only included a rather minor population, which is not representative enough for the entire population and might lead to biases in statistical results. However, our data were collected from several local hospitals and our results are also in line with several other studies. Therefore, although not nationwide data, our results could at least be representative of the northern part of Taiwan. Furthermore, most epidemiological studies have showed that type I FCoV is more prevalent than type II FCoV [12,18,27], which is consistent with our result that type I infection (21/47, 45%) was more common, and type II infection (9/47, 19%) and co-infection (9/47, 19%) were less common. For those co-infected individuals, whether the type I strain also dominates the infection in the cat host remains unknown and awaits further studies. In addition, after conducting a comparison with reference strains, we found that our type I isolated strains could be grouped with different strains isolated around the world, including Korea, Japan, Belgium, the US, the UK, Denmark, and the Netherlands, while type II strains were more aggregated and only grouped with other Taiwanese strains isolated 10 years ago. Our finding of a high level of variation among type I isolated strains is similar to that reported in studies of China in 2018 [16] and Taiwan in 2007 [21], but different from data presented in a study of Korea in 2009, in which type I strains were very aggregated.

## 4. Materials and Methods

### 4.1. Cat Specimens

In the period between September 2017 and January 2019 in Taiwan, 81 effusion specimens (41 ascites, 34 pleural effusion, 2 with both ascites and pleural effusion, 1 renal subcapsular fluid, 1 pericardial effusion, 1 thoracic cyst fluid, and 1 abdominal cyst fluid) from FIP-suspected cats submitted by local animal hospitals or National Taiwan University Veterinary Hospital for FCoV detection were recruited for this study. The signalments of these cats were recorded with as much detail as possible.

### 4.2. Indirect Immunofluorescence Assay

Our IFA protocol was a modified version of Lister et al. [10]. Briefly, the cell count of the sample was determined upon its arrival at our laboratory, and the sample was either concentrated or diluted to a concentration of 500,000 cells per 100 µL for further procedures. Only samples containing sufficient cells were included in this study. The sample was centrifuged using a cytospin centrifuge at 1000 rpm for 10 min, and the slide was fixed with 80% acetone at −20 °C for 10 min. Then, after slight air drying, the specimen dot was circled with a PAP pen. To label FCoV antigens, 10% normal goat serum (Jackson ImmunoResearch, West Grove, PA, USA) was added to the slide for 30 min for blocking at room temperature, and the mouse anti-FCoV N protein monoclonal antibody (#MCA2194, Bio-Rad Laboratories, Hercules, CA, USA) was added to the slide at a 1:400 dilution in 10% goat serum for 40 min as a primary antibody and washed with PBS three times. Then, to specifically label the marker, the goat anti-mouse IgG FITC conjugate (Jackson ImmunoResearch) was added at a 1:400 dilution in 10% goat serum for 30 min as a secondary antibody at room temperature in the dark. The slides were again washed three times with PBS and then sealed with mounting solution containing DAPI (Vectashield, Burlingame, CA), which labels DNA and can dye the nucleus of macrophages. Upon interpretation, macrophages were located under the phase-contrast imaging, and the presence of cytoplasmic fluorescence within macrophages was considered positive.

### 4.3. Viral RNA Extraction and RT-PCR

Viral RNA was extracted from samples using the PetNAD Nucleic Acid Co-Prep kit (GeneReach, Taichung, Taiwan), according to the manufacturer’s instructions. Next, cDNA was generated using M-MLV Reverse Transcriptase (Invitrogen, Carlsbad, CA, USA) with 1 µL of the gene-specific primer Iubs (for spike-targeting PCR) or random hexamers (for UTR-targeting PCR). These mixtures were heated to 65 °C for 5 min to denature the secondary structure of the viral RNA to enhance primer annealing, and a premixture for M-MLV-RT, which contains 4 µL of 5× FS buffer, 2 µL of 0.1 M DTT, 1 µL of 10 mM dNTP, and 1 µL of RNaseOUT, (Invitrogen), was added to each PCR tube. After mixing the contents of these tubes gently, they were heated to 32 °C for 2 min, and reverse transcription was performed at 37 °C for 30 min. A nested PCR targeting the 3′-UTR described by Herrewegh et al. [13] and the spike gene region described by Addie et al. [12] was carried out according to the protocol to differentiate the genotype I strains from the genotype II strains. A positive 3′-UTR should yield a 177-bp amplicon on electrophoresis, while type I strains could be distinguished by a 360-bp amplicon and type II strains yielded a 218-bp amplicon on electrophoresis.

### 4.4. Phylogenetic Analysis

The PCR products of the partial S gene were submitted to Tri-I Biotech Company (Taipei, Taiwan) for sequencing, and all nucleotide sequences generated in our study were submitted to GenBank. The sequences from this study, along with the reference strains (Table S1), were compiled and aligned using the Lasergene software package (DNASTAR, Madison, WI, USA) using the Clustal W method. Phylogenetic trees were constructed with the neighbor-joining method using MEGA software version 6.0. Bootstrap values were determined from 1000 replicates.

### 4.5. Statistical Analysis

The correlation of IFA positivity with sex, age, and breed was analyzed by Fisher’s exact test and the OR with a 95% CI. For comparisons of the two groups, a Student’s *t*-test was performed. The difference in values was considered statistically significant if the *p* value was smaller than 0.05. Figures were plotted using Prism 8 (GraphPad, San Diego, CA, USA).

## 5. Conclusions

This study recorded the current infection status of FCov in the northern part of Taiwan using both IFA and RT-PCR as detection methods and analyzed the sequences of the isolated FCoV strains. Our results revealed the significant correlation between the sex of sick cats, the low A/G ratio and the tendency of FCoV, and also reported the genetically divergent type I strains and the rather high prevalence of type II strains compared to other countries. This study gives us a profile of the current FCoV status and might provide an outline for future research on FCoV spike protein gene.

## Figures and Tables

**Table 1:** Immunofluorescence assay (IFA) detection of cat samples in this study.

IFA	Sex		Age (Month)		Breed	
	M	F	0~24	>24	Purebred	Non-Purebred
Positive	35	12	27	20	16	30
Negative	17	16	12	22	11	23

## Supplementary Materials

The following are available online at https://www.mdpi.com/2076-0817/9/9/698/s1. Table S1: FCoV reference sequences used in this study.
