# Supplementary material for: Detection of Feline Coronavirus in Feline Effusions by Immunofluorescence Staining and Reverse Transcription Polymerase Chain Reaction
Source: Pathogens. 2020 Aug 25;9(9):698. doi: 10.3390/pathogens9090698 (PMC7559213; doi:10.3390/pathogens9090698)
Supplement: Supplementary file 1 [file pathogens-09-00698-s001.zip › pathogens-884879-suppl.docx]

**Table 1.** FCoV reference sequences used in this study.

| **Strain** | **Country** | **Accession number** | **Genotype** |
| --- | --- | --- | --- |
| FCoV/NTU22/P/2004 | Taiwan | EU513381 | I |
| FCoV/NTU45/P/2005 | Taiwan | EU513382 | I |
| FCoV/NTU47/A/2006 | Taiwan | EU513383 | I |
| FCoV/NTU48/A/2007 | Taiwan | EU513384 | I |
| FCoV/NTU30/A/2004 | Taiwan | EU513385 | II |
| FCoV/NTU39/A/2005 | Taiwan | EU513386 | II |
| FCoV/NTU46/Ce/2006 | Taiwan | EU513387 | II |
| FCoV/NTU156/P/2007 | Taiwan | EU513388 | II |
| FCoV/NTU205/C/2007 | Taiwan | EU513389 | II |
| Cat 1 Karlslunde | Denmark | KX722530 | I |
| KU-2 | Japan | D32044 | I |
| F13-27 | Japan | EF408013 | I |
| UCD1 | Japan | AB088222 | I |
| F6-34-I | Japan | EF408019 | I |
| UG-FH8 | Belgium | KX722529 | I |
| Felis catus/  NLD/UU88/2010 | The Netherlands | KF530123 | I |
| UU23 | The Netherlands | GU553362 | I |
| UU10 | The Netherlands | FJ938059 | I |
| UU54 | The Netherlands | JN183883 | I |
| 08K-958 | Korea | JN654404 | I |
| 08K-420 | Korea | JN654401 | I |
| 08K-478 | Korea | JN654402 | I |
| 08K-559/II | Korea | JN654411 | II |
| 08K-609 | Korea | JN654412 | II |
| 08K-656 | Korea | JN654413 | II |
| FCoV C1Je | United Kingdom | DQ848678 | I |
| 26M | United Kingdom | KP143512 | I |
| 79-1683 | United Kingdom | X80799 | II |
| FIPV 79-1146 | United Kingdom | DQ010921 | II |
| Black | USA | EU186072 | I |
| RM | USA | FJ938051 | I |
| DF-2 | USA | DQ286389 | II |
